# Ankle-brachial index and eicosapentaenoic acid/arachidonic acid ratio in smokers with type 2 diabetes mellitus

**DOI:** 10.1186/s12971-016-0068-9

**Published:** 2016-01-28

**Authors:** Kenta Okada, Kazuhiko Kotani, Shun Ishibashi

**Affiliations:** Division of Endocrinology and Metabolism, Department of Internal Medicine, Jichi Medical University, 3311-1 Yakushiji, Shimotsuke-City, Tochigi 329-0498 Japan; Nasu Chuoh Hospital, 1453 Shimoishigami, Otawara-City, Tochigi 329-0436 Japan; Division of Community and Family Medicine, Jichi Medical University, 3311-1 Yakushiji, Shimotsuke-City, Tochigi 329-0498 Japan

**Keywords:** Arachidonic acid, Eicosapentaenoic acid, Peripheral arterial disease, Smoking

## Abstract

**Background:**

The ankle-brachial index (ABI) is an indicator of peripheral arterial damage and a low (ABI ≤ 1.0) or borderline (ABI = 1.00–1.09) value is associated with risk of cardiovascular disease events. A low ratio of serum eicosapentaenoic acid to arachidonic acid (EPA/AA) is also a risk factor for cardiovascular disease events. This study examined associations between the ABI and the EPA/AA ratio in smokers and non-smokers with type 2 diabetes mellitus (T2DM).

**Findings:**

Blood data including EPA, AA, and ABI were measured in smokers and non-smokers with T2DM enrolled at Jichi Medical University (*n* = 116, male 86 %, mean age 59 yr). The patients were classified into two groups according to their ABI level: <1.1 (low to borderline) or ≥1.1 (high). The EPA/AA ratio in smoking patients with ABI < 1.1 (*n* = 26; EPA/AA = 0.25) was significantly lower than in those with ABI ≥ 1.1 (*n* = 32; EPA/AA = 0.34; *p* = 0.03), but was not significantly different in non-smoking patients. The EPA/AA ratio was independently, significantly, and positively correlated with the ABI level (*β* = 0.41; *p* < 0.01) after adjusting for multiple variables only in smoking patients with T2DM.

**Conclusions:**

The EPA/AA ratio may be associated with subclinical peripheral arterial damage in smokers with T2DM. Further studies are warranted.

## Findings

### Background

Smokers have an increased risk of peripheral arterial damage, which is associated with cardiovascular disease (CVD) events in patients with diabetes mellitus (DM) [1]. Multi-faceted studies of subclinical peripheral arterial damage are thus important for prevention of CVD in patients with DM. Assessment of peripheral arterial damage is often conducted noninvasively using the ankle-brachial index (ABI) [2, 3]. Although ABI < 0.9 has diagnostic value for peripheral arterial disease (PAD) [2, 3], a low-to-borderline level of ABI (0.9–1.1) is also associated with increased risk of CVD events and related mortality [4, 5].

A low ratio of serum eicosapentaenoic acid to arachidonic acid (EPA/AA) has also been correlated with CVD events in patients with DM [6, 7]. An additional study reported that the EPA/AA ratio was positively associated with the ABI level in hospitalized patients, although it did not take into account smoking habits or DM pathology [8]. Recently, we reported that a low EPA/AA ratio is often seen in elderly patients with type 2 DM (T2DM), particularly in smokers [9]. However, the association between the ABI and EPA/AA ratio has not been elucidated; therefore, in the present study, we aimed to investigate the correlation between ABI and EPA/AA among smokers with T2DM.

### Subjects and methods

#### Study participants

A cross-sectional study of 116 smoking and non-smoking T2DM patients (2 groups of 50 men and 8 women, mean age 59 yr in each group) who visited the Jichi Medical University Hospital, Shimotsuke, Japan for more 1 yr was conducted from April to September 2014. Smoking habits were confirmed via self-reports. Current smoking status was designated as either smoking or not smoking. None of the patients were taking medications containing EPA or AA. We excluded patients with a history of CVD events, recent acute illness, systemic inflammatory disease, severe nephropathy (i.e., stage 3–5), liver dysfunction, type 1 DM, and PAD. The study was approved by the Jichi Medical University Ethical Committee and informed consent was obtained from all patients.

Hypertension was defined as systolic blood pressure (SBP) ≥ 140 mm Hg, diastolic blood pressure ≥ 90 mm Hg, and/or anti-hypertensive drug use [10]. Nephropathy was defined as a urinary albumin-to-creatinine ratio > 30 mg/g creatinine, above the micro albuminuria level [11].

#### Data collection

Fasting blood samples were collected at the outpatient clinic to measure levels of glucose, hemoglobin A1c (HbA1c), total cholesterol, triglycerides, high-density lipoprotein cholesterol, EPA, and AA. A high-performance liquid chromatography system (HLA-723G8; Tosoh Corp., Tokyo, Japan) was used to measure HbA1c. Serum lipids were extracted by Folch’s method and the fatty acids (internal standard, tricosanoic acid [C23:0]) were methylated with boron trifluoride and methanol. EPA and AA levels in the methylated fatty acids were then analyzed by gas chromatography (GC-2010; Shimadzu Corp., Kyoto, Japan) with a capillary column (TC-70; GL Sciences Inc., Tokyo, Japan).

The ABI was determined using a pulse pressure analyzer (model BP-203RPEIII; Omron Colin Corp., Tokyo, Japan) as previously described [12]. ABI was determined based on the SBP in both the upper (brachial arterial) and lower (tibial arterial) arteries [13] and was calculated by dividing the ankle SBP by the brachial SBP. The patient population was divided into two ABI groups (<1.1 and ≥1.1) based on previous studies [4, 14].

#### Statistical analysis

Smoking and non-smoking patients were matched for age and sex. Comparisons between smoking and non-smoking groups were conducted using an unpaired *t*-test or chi-square test. Differences in parameters between ABI groups were analyzed using an unpaired *t*-test or chi-square test. Correlations between absolute ABI and the other parameters (including the EPA/AA ratio) were examined using a Pearson’s correlation test and stepwise multiple regression analysis (SPSS software; SPSS Inc., IL, USA). All parameters were included in the stepwise multiple regression model except for the EPA, which was not included due to multicollinearity with the EPA/AA ratio and the stronger correlation of the EPA/AA ratio with the ABI. Parameters with skewed distributions were log-transformed prior to all analyses. A *p*-value of <0.05 was considered to be significant.

### Results

Clinical characteristics of the study population for the smoking and non-smoking groups are shown in Table 1. The HbA1c level and percentage with neuropathy complications were higher in the smoking group than in the non-smoking group.

**Table 1:** Clinical characteristics of the study population by current smoking habit

Parameter	Non-smoker (*n* = 58)	Smoker (*n* = 58)	*p*
Age, yr	59 ± 12	59 ± 10	0.91
Sex, male (%)	50 (86 %)	50 (86 %)	1.00
Body mass index, kg/m^2^	26.3 ± 4.4	26.2 ± 4.5	0.88
Systolic blood pressure, mm Hg	131 ± 14	131 ± 11	0.80
Diastolic blood pressure, mm Hg	77 ± 11	79 ± 10	0.43
Antihypertensive drugs, *n* (%)	33 (57 %)	43 (74 %)	0.05
Glucose, mg/dL	149 ± 62	139 ± 42	0.29
Hemoglobin A1c, %	7.2 ± 1.0	7.6 ± 1.0	0.045*
Insulin injection, *n* (%)	15 (26 %)	14 (24 %)	0.83
Retinopathy, *n* (%)	18 (31 %)	23 (40 %)	0.33
Neuropathy, *n* (%)	24 (41 %)	36 (62 %)	0.03*
Nephropathy, *n* (%)	19 (33 %)	25 (43 %)	0.25
LDL cholesterol, mg/dL	97 ± 31	95 ± 33	0.73
HDL cholesterol, mg/dL	60 ± 17	55 ± 16	0.13
Triglycerides, mg/dL	114 (73–160)	125 (91–173)	0.19
Statin drugs, *n* (%)	21 (36 %)	22 (38 %)	0.85
AA, μg/mL	173 (151–212)	181 (152–234)	0.49
EPA, μg/mL	60 (41–96)	52 (36–86)	0.28
EPA/AA ratio	0.37 (0.23–0.56)	0.29 (0.19–0.42)	0.08
Ankle-brachial index	1.14 ± 0.07	1.11 ± 0.09	0.07

Data are means ± standard deviations, medians (interquartile ranges), or numbers (%)

*LDL* low-density lipoprotein, *HDL* high-density lipoprotein, *AA* arachidonic acid, *EPA* eicosapentaenoic acid

* *p* < 0.05 for comparison between groups by smoking (*t*-test or chi-square test)

Two patients with ABI < 0.9 (the defined PAD level) were included in the smoking group. In the smoking group, patients with ABI < 1.1 had significantly lower EPA/AA ratios than did those with ABI ≥ 1.1, while the AA and EPA alone did not show clear differences between patients with ABI < 1.1 and ≥ 1.1. The other parameters were not statistically different between the groups (Table 2). In contrast, patients with ABI ≤ 1.1 had a significantly higher HbA1c than did those with ABI ≥ 1.1 in the non-smoking group. There were no statistical differences in the other parameters, including EPA/AA, with ABI levels in the non-smoking group.

**Table 2:** Clinical characteristics of the study population by ABI (<1.1 and ≥ 1.1)

	Non-smoker		*p*	Smoker		*p*
Parameter	ABI < 1.1 (*n* = 17)	ABI ≥ 1.1 (*n* = 41)		ABI < 1.1 (*n* = 26)	ABI ≥ 1.1 (*n* = 32)	
Age, yr	56 ± 14	60 ± 11	0.21	58 ± 10	60 ± 11	0.61
Sex, male (%)	15 (88 %)	35 (85 %)	0.77	22 (85 %)	28 (88 %)	0.75
Body mass index, kg/m^2^	26.5 ± 5.6	26.2 ± 3.9	0.77	25.5 ± 3.8	26.7 ± 5.0	0.31
Systolic blood pressure, mm Hg	133 ± 17	130 ± 13	0.45	131 ± 10	132 ± 12	0.77
Diastolic blood pressure, mm Hg	80 ± 13	76 ± 10	0.22	79 ± 9	78 ± 10	0.76
Antihypertensive drugs, *n* (%)	12 (71 %)	21 (51 %)	0.18	20 (77 %)	23 (72 %)	0.66
Glucose, mg/dL	157 ± 39	146 ± 70	0.55	137 ± 42	140 ± 43	0.74
Hemoglobin A1c, %	7.7 ± 1.2	7.0 ± 0.8	0.01*	7.6 ± 1.0	7.5 ± 1.0	0.82
Insulin injection, *n* (%)	7 (41 %)	8 (20 %)	0.09	3 (12 %)	11 (34 %)	0.06
Retinopathy, *n* (%)	6 (35 %)	12 (29 %)	0.65	9 (35 %)	14 (44 %)	0.48
Neuropathy, *n* (%)	10 (59 %)	14 (34 %)	0.08	10 (38 %)	15 (47 %)	0.52
Nephropathy, *n* (%)	5 (29 %)	14 (34 %)	0.73	16 (62 %)	20 (63 %)	0.94
LDL cholesterol, mg/dL	96 ± 38	97 ± 28	0.88	95 ± 39	94 ± 28	0.95
HDL cholesterol, mg/dL	61 ± 18	59 ± 16	0.68	52 ± 14	57 ± 18	0.23
Triglycerides, mg/dL	127 (79–217)	107 (69–144)	0.18	139 (116–233)	118 (83–159)	0.09
Statin drugs, *n* (%)	6 (35 %)	15 (37 %)	0.93	13 (50 %)	9 (28 %)	0.09
AA, μg/mL	176 (144–214)	173 (150–210)	0.51	191 (158–244)	180 (134–227)	0.18
EPA, μg/mL	64 (46–114)	60 (41–97)	0.26	49 (33–69)	68 (41–96)	0.07
EPA/AA ratio	0.44 (0.21–0.75)	0.37 (0.23–0.55)	0.56	0.25 (0.16–0.40)	0.34 (0.20–0.69)	0.03*
Ankle-brachial index	1.06 ± 0.04	1.17 ± 0.05	<0.01**	1.03 ± 0.06	1.18 ± 0.05	<0.01**

Data are means ± standard deviations, medians (interquartile ranges), or numbers (%)

*LDL* low-density lipoprotein, *HDL* high-density lipoprotein, *AA* arachidonic acid, *EPA* eicosapentaenoic acid

* *p* < 0.05, ** *p* < 0.01 for comparison between ABI groups (*t*-test or chi-square test)

Correlations of absolute ABI with the other parameters are listed in Table 3. Pearson’s correlation tests found that EPA and the EPA/AA ratio were significantly and positively correlated with ABI levels in the smoking group, while HbA1c was significantly and negatively correlated with ABI levels in the non-smoking group. Stepwise multiple regression analysis revealed that the EPA/AA ratio was independently, significantly, and positively correlated with ABI levels and that use of statin drugs was inversely correlated with ABI levels in the smoking group, while HbA1c was independently, significantly, and negatively correlated with ABI levels in the non-smoking group.

**Table 3:** Correlations between the absolute value of the ankle-brachial index and other parameters

	Non-smoker		Smoker	
Parameter	*r* (*p*)	*β* (*p*)	*r* (*p*)	*β* (*p*)
Age, yr	0.23 (0.09)	NE	0.06 (0.67)	NE
Sex, male (%)	0.14 (0.31)	0.19 (0.12)	0.03 (0.81)	NE
Body mass index, kg/m^2^	−0.07 (0.61)	NE	0.05 (0.70)	NE
Systolic blood pressure, mm Hg	−0.05 (0.71)	NE	−0.04 (0.78)	NE
Diastolic blood pressure, mm Hg	−0.11 (0.41)	NE	−0.13 (0.35)	NE
Antihypertensive drugs, *n* (%)	−0.15 (0.27)	NE	−0.10 (0.45)	NE
Glucose, mg/dL	−0.16 (0.25)	NE	0.03 (0.85)	NE
Hemoglobin A1c, (%)	−0.46 (<0.01**)	−0.45 (<0.01**)	0.01 (0.95)	NE
Insulin injection, *n* (%)	−0.21 (0.12)	NE	0.19 (0.14)	0.26 (0.06)
Retinopathy, *n* (%)	−0.02 (0.91)	NE	−0.08 (0.54)	NE
Neuropathy, *n* (%)	−0.18 (0.18)	NE	−0.04 (0.79)	NE
Nephropathy, *n* (%)	0.08 (0.58)	NE	−0.05 (0.73)	NE
LDL cholesterol, mg/dL	−0.01 (0.95)	NE	−0.05 (0.72)	NE
HDL cholesterol, mg/dL	0.06 (0.67)	NE	0.22 (0.10)	NE
Triglycerides, mg/dL	−0.22 (0.11)	−0.16 (0.17)	−0.16 (0.27)	NE
Statin drugs, *n* (%)	−0.12 (0.37)	NE	−0.15 (0.26)	−0.31 (0.03*)
AA, μg/mL	0.04 (0.78)	–	−0.13 (0.32)	–
EPA, μg/mL	−0.19 (0.16)	–	0.28 (0.04*)	–
EPA/AA ratio	0.05 (0.73)	NE	0.31 (0.02*)	0.41 (<0.01**)

*LDL* low-density lipoprotein, *HDL* high-density lipoprotein, *AA* arachidonic acid, *EPA* eicosapentaenoic acid, *NE* not extracted; −: not used in the analysis. Data are Pearson’s *r* correlation coefficients and standardized *β* coefficients from stepwise multiple regression analysis

* *p* < 0.05, ** *p* < 0.01

### Discussion

We found that the EPA/AA ratio was lower in patients with ABI < 1.1 than in those with ABI ≥ 1.1, with a positive correlation between the EPA/AA ratio and ABI in smokers with T2DM. To our knowledge, a relationship between the EPA/AA ratio and ABI level among smokers or non-smokers with T2DM has not previously been reported. Accordingly, the results of this study suggest that the EPA/AA ratio may be associated with subclinical PAD in smokers with T2DM.

By contrast of n-3 fatty acids, particularly EPA, n-6 fatty acids, particularly AA, impair peripheral arteries by creating vasoactive oxygen radicals and promoting cyclooxygenase metabolism [15, 16]. In smokers, a delayed conversion of AA to eicosanoids is known [17–19], this may result in endothelial dysfunction in peripheral arteries [20]. This is a possible mechanism for a positive correlation between the EPA/AA ratio and ABI levels in smokers with DM, although this requires further research.

Based on the results of our stepwise regression model, ABI was inversely associated with the use of statin drugs in the smoking group. Statins are often prescribed for patients with DM, particularly when the patients may have complications of DM. As a result, patients with subclinical PAD may use statins. This is only a hypothesis; however, similar results were reported in a previous study [21].

The present study had certain limitations. It was a cross-sectional study with a small sample size; thus, further intervention and a placebo control study are needed. Additionally, as there was no information regarding dietary fish consumption, smoking dose, or smoking duration (e.g., the Brinkman index), and because the cigarette type was not investigated in detail, these factors could not be considered in our analysis.

### Conclusions

In summary, the EPA/AA ratio may be associated with subclinical PAD in smokers with T2DM. The utility of measuring the EPA/AA ratio along with ABI should be further investigated in future studies.

## Abbreviations

AA: arachidonic acid
ABI: ankle-brachial index
CVD: cardiovascular disease
DM: diabetes mellitus
EPA: eicosapentaenoic acid
HbA1c: hemoglobin A1c
PAD: peripheral arterial disease
SBP: systolic blood pressure
T2DM: type 2 diabetes mellitus
